# Supplementary material for: The interactome of intact mitochondria by cross-linking mass spectrometry provides evidence for coexisting respiratory supercomplexes
Source: Mol Cell Proteomics. 2017 Dec 8;17(2):216–32. doi: 10.1074/mcp.RA117.000470 (PMC5795388; doi:10.1074/mcp.RA117.000470)
Supplement: Supplemental Data [file supp_RA117.000470_133922_0_supp_23978_fzffwf.zip › spectra_annotation/mito_spectra_annotation/165-2-34-1-5-1.pdf]

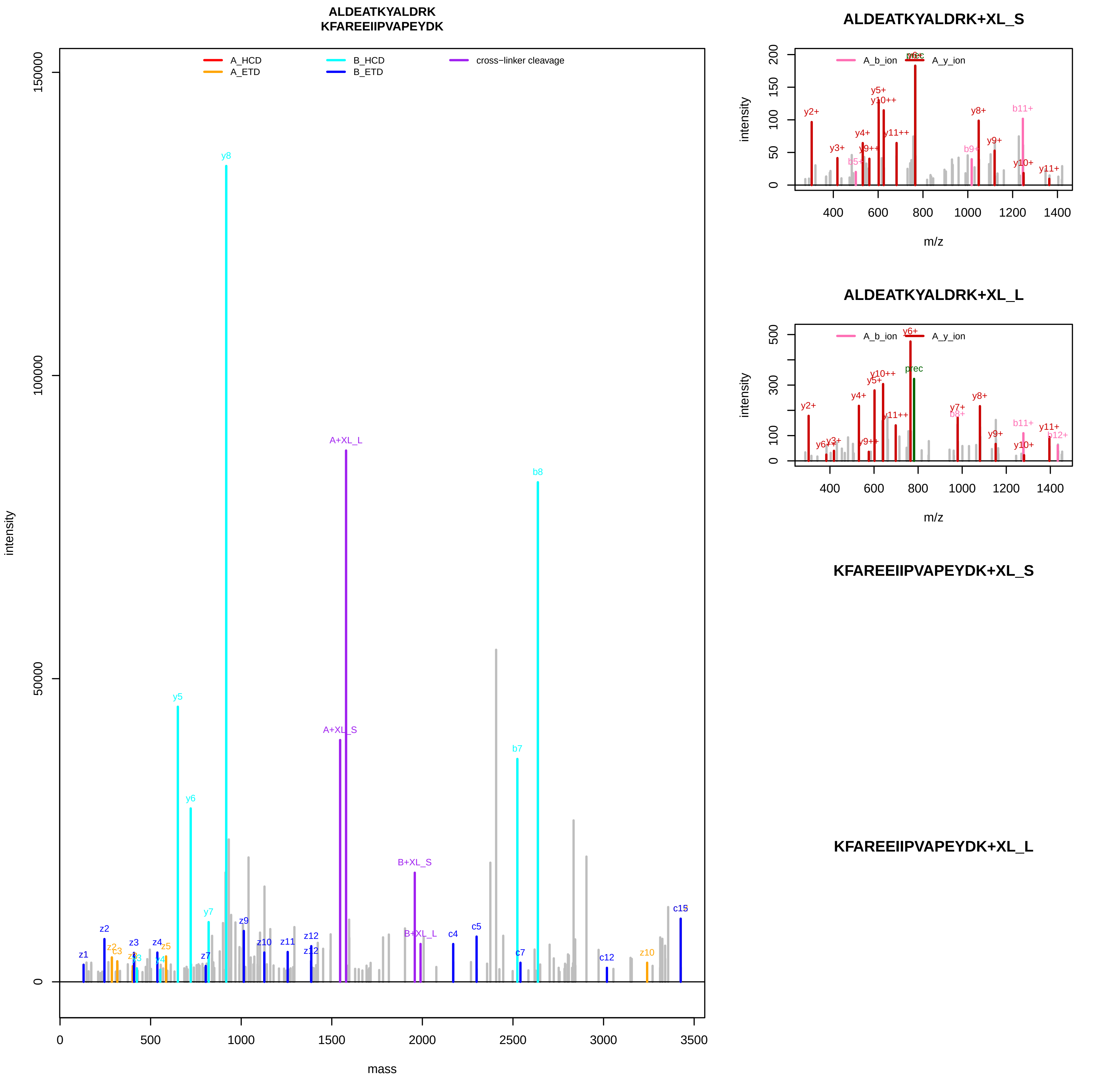
ALDEATKYALDRK
KFAREEIIPVAPEYDK
A_HCD
A_ETD
B_HCD
B_ETD
cross−linker cleavage
intensity
mass
y8
A+XL_L
b8
y5
A+XL_S
b7
y6
B+XL_S
y7
z9
c15
z2
z3
z4
z5
c5
c4
B+XL_L
z10
z11
z12
z1
z7
c7
c12
z10
ALDEATKYALDRK+XL_S
A_b_ion
A_y_ion
prec
m/z
ALDEATKYALDRK+XL_L
KFAREEIIPVAPEYDK+XL_S
KFAREEIIPVAPEYDK+XL_L
